# Protective Efficacy of a Modified Vaccinia Ankara-Based Vaccine Against Zika

**DOI:** 10.3390/vaccines14030252

**Published:** 2026-03-10

**Authors:** Leidi Carvajal Aristizabal, Ivanna Hoyos Ramírez, Erwin Camacho, Daniel Maldonado, Esteban Marín, Juan Pablo Hernández-Ortiz, Jorge E. Osorio

**Affiliations:** 1 VaxThera, Medellín 050031, Colombia; lycarvajal@vaxthera.com (L.C.A.); ighoyos@vaxthera.com (I.H.R.); dmaldonadop@vaxthera.com (D.M.); emarinre@vaxthera.com (E.M.); jphernandezo@vaxthera.com (J.P.H.-O.); 2 One Health Genomic Laboratory, Department of Materials and Nanotechnology, Facultad de Minas, Universidad Nacional de Colombia, Sede Medellín, Medellín 050034, Colombia; 3 Department of Pathobiological Sciences, School of Veterinary Medicine, University of Wisconsin-Madison, Madison, WI 53706, USA

**Keywords:** Zika virus, vaccines, flavivirus, Modified Vaccinia Ankara virus, immunity, cell-mediated immunity, neutralizing antibodies, mice

## Abstract

**Background:** Zika virus (ZIKV) is a mosquito-borne flavivirus associated with severe neurological disease, including congenital Zika syndrome (CZS) following utero infection and Guillain–Barré syndrome in adults. The 2015–2016 epidemic in the Americas highlighted the profound maternal and neonatal consequences of ZIKV infection. Although reported transmission has declined, ongoing circulation of competent vectors and population susceptibility sustain a substantial risk of future outbreaks, underscoring the need for effective vaccines. **Methods:** We developed a recombinant Modified Vaccinia Ankara (MVA)-based vaccine candidate expressing the ZIKV pre-membrane (prM) and envelope (E) proteins and evaluated its immunogenicity and protective efficacy in interferon receptor-deficient AG129 mice. **Results:** Vaccination induced strong humoral and cellular immune responses and conferred significant protection against viral replication in key target organs, including the brain and testes, following ZIKV challenge. **Conclusions:** These preclinical findings support further development of this MVA-based ZIKV vaccine as a promising strategy to prevent ZIKV infection and its associated neurological complications.

## 1. Introduction

Zika virus (ZIKV) is a mosquito-borne flavivirus that emerged as a major global health threat following large outbreaks in the Americas and Southeast Asia, culminating in the World Health Organization’s declaration of a Public Health Emergency of International Concern in 2016 [1,2]. During this epidemic, ZIKV transmission was causally linked to severe neurological complications, most notably congenital Zika syndrome (CZS) [3]. CZS is characterized by microcephaly, intracranial calcifications, ventriculomegaly, and other profound abnormalities of the central nervous system [4]. In adults, ZIKV infection has also been associated with Guillain–Barré syndrome, further underscoring the virus’s neurotropic potential [5]. Although reported ZIKV incidence has declined in recent years, the virus remains endemic in multiple tropical and subtropical regions. The risk of outbreaks persists due to a convergence of factors, including climate change, rapid urbanization, increased global mobility, and the continued presence of competent mosquito vectors such as *Aedes aegypti* and *Aedes albopictus* [6]. Notably, ZIKV also exhibits non-vector transmission routes, including sexual transmission and vertical transmission from mother to fetus, amplifying its public health impact [7]. Together, these factors underscore the urgent need for safe and effective vaccines capable of preventing ZIKV infection and its associated complications [8,9].

To date, multiple vaccine platforms have been explored with varying success in preclinical and clinical studies, including DNA vaccines, mRNA vaccines, viral-vectored vaccines, and virus-like particle (VLP) based approaches [8,10]. Among viral vectors, Modified Vaccinia Ankara (MVA) has emerged as a particularly promising platform due to its favorable safety profile, strong immunogenicity, and capacity to accommodate large and complex heterologous antigens [9,11]. MVA is a highly attenuated poxvirus that is replication-deficient in mammalian cells, making it suitable for use in immunocompromised populations. Importantly, MVA-based vaccines have demonstrated clinical success, including licensed vaccines for smallpox and mpox [12].

In this study, we describe the development and comprehensive characterization of a recombinant MVA-based vaccine candidate expressing the ZIKV pre-membrane (prM) and envelope (E) proteins (rMVA-ZIKV). These structural antigens were selected based on their essential roles in viral particle maturation and host cell entry, and their established function as primary targets of neutralizing antibody [3,13]. During virion maturation, prM undergoes proteolytic cleavage, while the E protein mediates receptor binding and membrane fusion, rendering both proteins critical targets for immune recognition. Their inclusion in the vaccine design aims to mimic key aspects of natural infection and to elicit coordinated humoral and cellular immune responses required for effective viral control and durable protection.

Using a robust preclinical model, we demonstrate that this rMVA-ZIKV vaccine candidate induces strong humoral and cellular immune responses and provides significant protection against ZIKV challenge with strains associated with severe neurological disease. We present a comprehensive evaluation of the vaccine, including its production and in vitro characterization, immunogenicity, and protective efficacy in AG129 mice, a model previously characterized and used by our group [10,14,15]. In particular, we assessed the relative contributions of antibody-mediated and cell-mediated immunity to protection against challenge with distinct ZIKV strains, including French Polynesian and Puerto Rican isolates obtained during microcephaly-associated outbreaks [16]. Collectively, these findings support the further development of rMVA-ZIKV as a promising vaccine candidate to prevent ZIKV infection and its devastating neurological sequelae.

## 2. Materials and Methods

### 2.1. Cells and Viruses

For rMVA-ZIKV production, the DF-1 cell line permissive model for MVA replication, was used to generate and propagate the recombinant virus carrying the ZIKV antigens. DF-1 cells obtained from IDT Biologika (IDT, Rockville, MD, USA. DF-1 RCB TG2020 Lot. 291-C194 #024) were adapted to Virus Production Serum-Free Medium (VP-SFM Gibco, Thermo Fisher Scientific, Waltham, MA, USA; Cat. No. 12559019) supplemented with 2% fetal bovine serum (FBS, Gibco, Thermo Fisher Scientific, Waltham, MA, USA; Cat. No. 10437-028) and 1% GlutaMAX (Gibco, Thermo Fisher Scientific, Waltham, MA, USA; Cat. No. 35050-061). To produce VLPs encoding ZIKV proteins (ZIKV-VLPs), the HEK-293 cell line (ECACC, Cat. No. 85120602; Salisbury, UK) was maintained in DMEM supplemented with 10% FBS and 1% GlutaMAX. A confluent monolayer of HEK-293 cells in T-75 flasks was transiently transfected as described below.

Vero cells obtained from ATCC (ATCC: CCL-81; Manassas, VA, USA) were employed for propagation of the ZIKV French Polynesia strain (H/PF/2013) and the prototype strain isolated in Puerto Rico (PRVABC-59). Vero cells were cultured in Dulbecco’s Modified Eagle Medium (DMEM, Gibco, Cat. No. 12100-061) supplemented with 5% fetal bovine serum, 1% GlutaMAX, and 1% antibiotic-antimycotic solution (Anti-Anti, Gibco, Thermo Fisher Scientific, Waltham, MA, USA; Cat. No. 15240062). Viral stocks were prepared by inoculating cells at a multiplicity of infection (MOI) of 0.2 and collecting supernatants at 48 and 96 h postinfection. Virus-containing media were clarified by centrifugation at 3200× *g* for 10 min using a Sorvall ST 16R centrifuge (Thermo Scientific, Waltham, MA, USA), titrated, and stored at −80 °C until use.

Throughout the study, all cell lines were maintained at 37 °C with 5% CO_2_ and seeded in 96- or 24-well plates, depending on the type of experiment, and in flasks for viral stock or VLPs production.

### 2.2. Expression Cassette Designs

The recombinant expression cassette was engineered to insert the ZIKV prM and E genes into the thymidine kinase (TK) locus of the Modified Vaccinia Ankara (MVA) genome. A pBSK (+) plasmid containing the prM/E sequence was used as the transfer vector. A total of 620 complete ZIKV genome sequences available in the NCBI database as of November 2021 were curated and translated into their corresponding protein sequences. The precursor membrane (prM) and envelope (E) structural proteins were analyzed using Shannon entropy, Simpson diversity, and Wu–Kabat variability indices to quantify amino acid variability at each residue position. The E protein exhibited variability ranging from 5% to 18% at specific amino acid sites, whereas the prM protein displayed variability between 5% and 10%. Based on these analyses, a consensus sequence was generated to represent the most frequent amino acids across all sequence pools. BLAST (BLASTP 2.16.0+) analysis of this consensus sequence identified a corresponding entry in GenBank (accession number QOF88708.1), which was subsequently used for vaccine candidate design.

The prM/E genes were placed under the control of a synthetic early/late (S E/L) promoter, generating the plasmid pBSK-ZIKV/GFP.DR. This construct included DNA sequences homologous to the left (TKL) and right (TKR) arms of the TK locus to promote recombination in MVA-infected cells. The enhanced green fluorescent protein (eGFP) gene was inserted downstream of E under control of the P11 late promoter and separated by a short direct repeat (DR) sequence, enabling its subsequent excision by intramolecular recombination to produce the final recombinant virus, rMVA-ZIKV, lacking eGFP. This design enables initial selection and monitoring of recombinant virus formation through GFP expression, followed by the removal of the marker to obtain a marker-free rMVA-ZIKV backbone suitable for vaccine development.

### 2.3. Production of Recombinant Viruses

To produce the rMVA-ZIKV vaccine, the previously synthesized plasmid was transfected into DF-1 cells using FuGENE^®^ HD Transfection Reagent (Promega, Madison, WI, USA; Cat. No. E2311), following infection with the parental Modified Vaccinia Ankara virus (Parental MVA). After 48 h, fluorescence of the marker incorporated into the plasmid sequence was detected, confirming successful recombination. The infected monolayer was then collected, and several rounds of plaque selection were performed to isolate the rMVA-ZIKV and eliminate the residual parental MVA. Once the absence of parental MVA was confirmed by PCR, serial passages were initiated to eliminate the fluorescence of the recombinant clone. After multiple passages, non-fluorescent clones lacking the GFP gene were selected by limiting dilution, isolating clones without fluorescence but with a typical cytopathic effect (CPE). To verify the identity of the recombinant obtained, the absence of the fluorescence gene and the integrity of the expression cassette were verified by PCR.

To continue serial expansion and generate the seed stock, the selected clone was scaled up by increasing the growth area. Infected cells were collected and homogenized using a dounce homogenization followed by sonication. The virus was subsequently purified by ultracentrifugation through a 36% (*w*/*v*) sucrose cushion (1:1 virus-to-sucrose ratio) at 25,862× *g* for 1.13 h at 4 °C and stored at −80 °C until further use. The resulting seed stock was used to generate the subsequent virus stocks for in vivo studies and in vitro characterization, following the same purification steps. Each stock produced was characterized by PCR to verify the integrity of the cassette containing the ZIKV antigens, by Western blot to confirm antigen expression, and an immunoassay titration to determine the viral titer (PFU/mL) after purification.

For ZIKV-VLPs obtention, HEK-293 cells were transiently transfected using Opti-MEM™ (Gibco, Thermo Fisher Scientific, Waltham, MA, USA; Cat. No. 22600-134) and FuGENE^®^ HD Transfection Reagent following the manufacturer’s instructions. A cytomegalovirus promoter–driven plasmid encoding the ZIKV prM and E proteins (pCMV-prM/E) was used for transfection. The plasmid construct and purification procedures followed a previously described protocol [15].

Culture supernatants were harvested at 72 h post-transfection (hpt) and clarified by centrifugation at 15,000× *g*. Clarified supernatants were then layered at a 1:1 ratio onto a 20% (*w*/*v*) sucrose cushion and purified by ultracentrifugation at 112,000× *g* for 3.5 h at 4 °C. The resulting pellet containing ZIKV-VLPs was resuspended in phosphate-buffered saline (PBS, pH 7.2) and stored at −80 °C until further use. Total protein concentration was determined using the Pierce™ BCA Protein Assay Kit (Thermo Scientific, Waltham, MA, USA; Cat. No. 23227). For vaccination, the ZIKV-VLP formulation was prepared with 2% (*w*/*v*) aluminum hydroxide as adjuvant.

### 2.4. Immunoassay Titration

Plaque-forming units (PFU) were determined by immunoassay titration using a 1:10 serial virus dilution system in DF-1 cells, performed in triplicate per sample in 96-well plates. Infected cells were incubated for 36 h and then fixed with paraformaldehyde. Subsequently, cells were incubated with a primary anti-vaccinia virus antibody (Abcam, Cambridge, UK; Cat. No. AB35219) diluted 1:1000 in 5% non-fat milk in PBST for 2 h at room temperature, followed by incubation with an HRP-conjugated secondary antibody (Thermo Scientific, Waltham, MA, USA; Cat. No. 31460) under the same conditions but protected from light. The signal was developed at room temperature for 40 min using an AEC solution containing hydrogen peroxide. Resulting plaques were captured using the ImmunoSpot system (CTL, Cleveland, OH, USA) and quantified with the Viridot package (v1.0) in RStudio (v2025.05.1+513, R v4.5.1). The final titers were determined from plaque counts and reported as PFU/mL.

### 2.5. Mouse Studies

To evaluate whether our rMVA-ZIKV vaccine candidate induces ZIKV-specific immunity and a protective profile, we used the AG129 mouse strain, a model previously described and validated by us [10,17]. This model was utilized to assess the efficacy and immune responses elicited by vaccination with the rMVA-ZIKV construct.

Groups of 4- to 6-week-old AG129 mice (*n* = 13) were immunized via intramuscular (IM) injection in the thigh muscles with either one or two doses of the rMVA-ZIKV (1 × 10^8^ PFU/dose), two doses of ZIKV VLPs (ZIKV-VLP; 2 µg/dose) as a positive control, or phosphate-buffered saline (PBS) as a negative control (mock), in a 50 µL volume. Booster doses were administered three weeks after initial (prime) vaccination.

On day 34 post-prime vaccination, seven animals per group were euthanized for spleen collection to perform adoptive transfer studies using single cell splenocytes suspensions. The remaining animals were challenged on day 35 with 1 × 10^2^ PFU of the ZIKV H/PF/2013 strain via IM route and monitored daily for two weeks to assess bodyweight changes and clinical signs. Animals were humanely sacrificed either at the study endpoint or earlier if euthanasia criteria were met.

Blood samples were collected on days 20 (pre-boost), 28 and 34 (pre-challenge) to obtain serum for: (i) ZIKV-specific neutralizing antibody titration via foci reduction neutralization test (FRNT) and (ii) passive transfer experiments. Additional blood samples were collected at 3 days post-challenge and at the study endpoint for viremia quantification by plaque assay. At the endpoint, brain and testicular tissues were harvested for viral load analysis by plaque assay.

To dissect the contribution of cellular and humoral immunity to vaccine-induced protection, adoptive and passive transfer studies were conducted. For the adoptive transfer, 6-week-old AG129 mice (*n* = 5) were retro-ocularly inoculated with 1 × 10^7^ splenocytes isolated from vaccinated donors (rMVA-ZIKV prime, rMVA-ZIKV prime and boost, ZIKV VLP and PBS) sacrificed one day before ZIKV challenge. Pooled splenocytes obtained from each experimental group were used for adoptive transfer. Sixteen hours post-transfer, mice were challenged with 1 × 10^2^ PFU of the H/PF/2013 strain via IM route and monitored daily for two weeks. Blood samples were collected at 3 dpc and at the study endpoint for viremia quantification, and brain and testicular tissues were harvested for viral load determination by plaque assays.

For passive transfer, pooled sera collected on days 28 and 34 from vaccinated mice were administered intraperitoneally (IP) to 6-week-old AG129 mice (*n* = 6). Sixteen hours post-inoculation, mice were challenged with 1 × 10^4^ PFU of the PRVABC59 ZIKV strain via IM route and monitored for two weeks. Blood samples were collected at 3 dpc and at the endpoint for viremia analysis, and brain and testicular tissues were also collected for viral load quantification by plaque assays. Tissues (brain, testes, liver, kidney, and spleen) were collected for histopathological examination by a pathologist who was blinded to the clinical outcomes in the study. Histopathological alterations were scored using a semi-quantitative scale ranging from 0 (no identified changes) to 4 (severe lesions), following established grading criteria. For each animal and tissue type, composite severity scores were calculated by summing individual parameter scores to enable comparisons between groups.

### 2.6. Neutralization Assay

To evaluate the neutralizing antibody induction capacity in each experimental group, the Zika virus (ZIKV) strain PRVABC-59, previously isolated and titrated, was used. Collected mouse serum samples were diluted 1:4 in dilution plates with DMEM medium. The ZIKV strain PRVABC-59 virus was then prepared in DMEM supplemented with 5% FBS and added to the wells containing the diluted sera in a 1:1 ratio. Negative controls (no virus) and wells with virus without serum samples were included. The plates were incubated at 37 °C with 5% CO_2_ for 1 h to allow neutralization. Then, 50 µL of each dilution was transferred, in duplicate, to 96-well plates previously seeded with Vero CCL-81 cells. The cells were incubated with the samples at 37 °C for 2 h, shaking every 20 min. After incubation, 150 µL of overlay containing carboxymethylcellulose (CMC, Sigma-Aldrich, St. Louis, MO, USA; Cat. No. 9004-32-4) (DMEM with 1.5% CMC and 2% FBS) was added. The plates were incubated for 48 h before starting the immunoassay, using an anti-flaviviridae primary antibody followed by an anti-mouse secondary antibody. 1-Step™ TMB-Blotting Substrate Solution (Thermo scientific, Waltham, MA, USA; Cat. No. 34018) was used for plaque chromogenic detection, and the plates were scanned using an ELISpot reader. Plaque quantification was performed using Viridot software in RStudio. Finally, the percentage of neutralization was calculated for each sample.

### 2.7. Plaque Assay

For titration of stocks and assessment of viral load in serum and tissues, the sample was serially diluted (10-fold dilutions), and 100 µL per well was used to infect 24-well plates monolayers of Vero cells at 37 °C for 2 h, shaking every 20 min. The monolayer was covered with a mixture of DMEM and 1.5% CMC supplemented with 2% FBS, after infection. Cells were maintained at 37 °C, 5% CO_2_ for 4 days for plaque development. Before plaque counting, cells were fixed with 10% formaldehyde for 30 min and stained with 1% crystal violet in 20% ethanol.

### 2.8. Molecular Testing (PCR)

To characterize the rMVA-ZIKV clones, endpoint PCR was performed. Two sets of main primers were used: primers specific for the TK recombination site were employed to assess the presence of the parental MVA (TKR_Forward, CGTGTGTAGAAAGTGTTACGTCG, TKL_Reverse, ACGGTTTATCTAACGACACAACATCC, 2255 bp), and a second primer set was used to validate the integration of the cassette of interest at the prM-E target sequence (zk_prM_E_Forward, ATCTCATTCCCGACCACCCT, zk_Prm_E_Reverse, AATCAGACGCCACCTAACGC, 1926 bp). Additionally, to confirm GFP absence, another set was employed (TKR2_Forward, GGTGGTAAAACTAACTGCTGTGTGT, GFP_Reverse, GCCGTCGTCCTTGAAGAAGA, 2772 bp). The amplification was done using the enzyme OneTaq^®^ Quick-Load^®^ DNA Polymerase (NEB, Ipswich, MA, USA; Cat. No. M0509L). The resulting fragments were evaluated by electrophoresis in 1.0% agarose gel in TAE buffer at 100 V for 60 min.

To quantify ZIKV in mice after viral challenge, real-time PCR was performed using primers specific for the NS4 region (qZIKV_NS4_Forward, CAGCTGGCATCATGAAGAAYC, qZIKV_NS4_Reverse, CACCTGTCCCATCTTTTTCTCC, ZIKV_NS4_Probe, FAM-CYGTTGTGGATGGAATAGTGG-BHQ1). The enzyme iTaq Universal One-Step RT-qPCR Kit (BioRad, Hercules, CA, USA; Cat. No. M0509L) was used with a thermal profile of 50 °C for 10 min, 95 °C for 3 min and 35 cycles of 95 °C for 15 s, 55 °C for 15 s. For the quantification curve, previously produced and quantified RNA standards were used.

### 2.9. Antigen Detection

Antigen expression of ZIKV Pre-Membrane (PrM) and Envelope (E) proteins encoded in the recombinant cassette was confirmed by Western blot (WB) and immunofluorescence assays. WB was performed using a flavivirus envelope recombinant antibody ([4G2], Thermo Scientific, Cat. No. MA5-47848) for E protein detection and a Zika virus PrM antibody (Genetex, Irvine, CA, USA; Cat. No. GTX133305) for PrM protein detection as primary antibodies. Protein separation was carried out by electrophoresis under native or denaturing conditions in a 4–20% gradient acrylamide gel employing a Mini-Protean System (BioRad), followed by transfer to nitrocellulose membranes. Membranes were blocked with 5% non-fat milk in PBST for 1 h at room temperature. Primary antibodies were diluted in blocking buffer at 1:1000 for E detection and 1:2000 for PrM detection and incubated overnight at 4 °C with gentle agitation. An HRP-conjugated secondary antibody (Thermo Scientific, Waltham, MA, USA; Cat. No. 31460) was added at a 1:5000 dilution and incubated for 2 h at room temperature with gentle shaking in the dark. Signal development was performed using a TMB (3,3′,5,5′-Tetramethylbenzidine) solution for revealing, and imaging was acquired with the ChemiDoc System (Bio-Rad, Hercules, CA, USA).

For immunofluorescence assays, DF-1 cells were seeded in 24-well plates the day before infection and subsequently infected with the corresponding viral stock. Twelve hours post-infection, cells were fixed with paraformaldehyde and permeabilized with methanol. After washing, cells were incubated for 3 h at room temperature with the flavivirus envelope recombinant antibody (4G2) for envelope protein detection. Subsequently, cells were washed again and incubated for 2 h in darkness at room temperature with a fluorescein-conjugated secondary antibody 1:1000 diluted (Invitrogen, Thermo Fisher Scientific, Waltham, MA, USA; Cat. No. F2765). After incubation, plates were carefully washed to remove residual fluorescence and visualized under the microscope using the EVOS Cell Imaging System (Thermo Scientific, Waltham, MA, USA). Resulting representative images were captured as evidence.

### 2.10. Statistics

Survival rates were compared using the log-rank (Mantel–Cox) test. Bodyweight changes were analyzed by two-way analysis of variance (ANOVA) with an uncorrected Fisher post hoc test. Log-transformed viral loads and FRNT50 titers were evaluated using one-way ANOVA followed by Tukey’s multiple comparison test. Total histopathology scores were analyzed using the non-parametric Kruskal–Wallis test. Statistical analysis was performed in GraphPad Prism v10.6.1 software (Boston, MA, USA). Statistical significance was defined as *p* < 0.05 (* *p* < 0.05, ** *p* < 0.01, *** *p* < 0.001, **** *p* < 0.0001).

## 3. Results

### 3.1. Production and In Vitro Characterization of rMVA-ZIKV Vaccine

**Figure 1:**
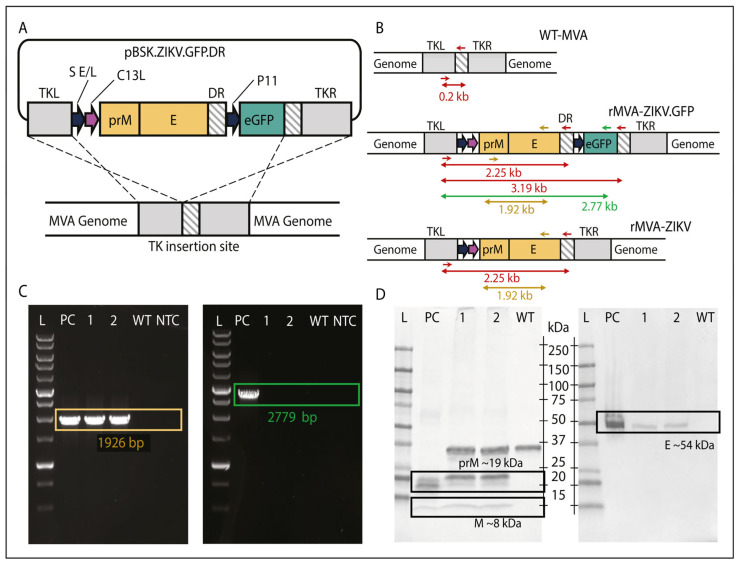
Overall process for the design, generation, and characterization of the rMVA-ZIKV vaccine candidate: (**A**) Schematic representation of the shuttle vector, showing the TK insertion site (gray), ZIKV prM/E genes (gold), and the eGFP marker (green). The vector also includes a duplicated repeat region (diagonal grey shading) flanking the eGFP cassette to enable its subsequent excision. (**B**) General workflow for the generation of recombinant viruses. The wild-type MVA (WT-MVA) is used as the backbone to generate the initial eGFP-expressing recombinant (rMVA-ZIKV.GFP). From this intermediate, the final recombinant lacking the fluorescent marker (rMVA-ZIKV) is obtained. Primer binding sites used for clone characterization are indicated by arrows: red for TK region, gold for the ZIKV prM/E region, and green for the eGFP region. (**C**) PCR amplification to confirm the presence of the prM/E genes (1.92 kb; gold box) and to verify the absence of the eGFP marker (2.77 kb; green box). (**D**) Expression evaluation by Western blot analysis detecting pre-Membrane (prM: ~19 kDa), Membrane (M: ~8–12 kDa) and Envelope (E: ~54 kDa) proteins. Lanes from left to right in both PCR and Western blot analyses correspond to: DNA length/molecular weight ladder (L), positive control (PC), rMVA-ZIKV seed stocks (1 and 2), WT-MVA (WT), and non-template control (NTC).

Transfection of MVA-infected DF-1 cells with the shuttle vector pBSK.ZIKV.GFP.DR, encoding the ZIKV prM/E structural genes (Figure 1A), resulted in the effective expression of the GFP fluorescent marker, confirming the generation of the recombinant rMVA-ZIKV, denoted as rMVA-ZIKV.GFP (Figure 1B and Figure S1A). The recombination cassette was designed to include a self-deleting marker-gene feature (a TK flank double repeat region) that allowed the removal of the GFP marker from the recombinant virus through intramolecular homologous recombination events occurring after several passages (Figure 1B). After eight serial passages and subsequent isolation by limiting dilution, non-fluorescent viral clones containing ZIKV prM/E genes (rMVA-ZIKV) were obtained. The loss of the marker-gene was confirmed by PCR amplification targeting both the recombination flanks and the ZIKV genes within the rMVA genome, with no signal of the GFP gene (Figure 1C). Additionally, plaque formation on DF-1 cells revealed cytopathic effects without fluorescence, confirming the successful removal of the GFP gene (Figure S1B).

The selected recombinant clone was subsequently expanded and purified to generate a master seed stock (MSS), which was thoroughly characterized by next-generation sequencing (NGS) to confirm the identity, integrity, and genetic stability of the recombinant virus. NGS analysis confirmed the correct insertion, orientation, and sequence fidelity of the ZIKV prM/E expression cassette within the rMVA genome, with no evidence of mutations, deletions, or genomic rearrangements across the analyzed passage series. These findings demonstrate the genetic stability of the construct and the consistent maintenance of the recombinant cassette throughout clone selection, seed stock generation, and serial viral propagation. Subsequently, the generated virus stock was characterized to assess the expression of ZIKV prM and E proteins by Western blot (Figure 1D) and immunofluorescence (Figure S1C), confirming the successful generation of rMVA-ZIKV.

### 3.2. rMVA-ZIKV Vaccine Candidate Is Immunogenic and Protects from ZIKV Challenge in AG129 Mice

**Figure 2:**
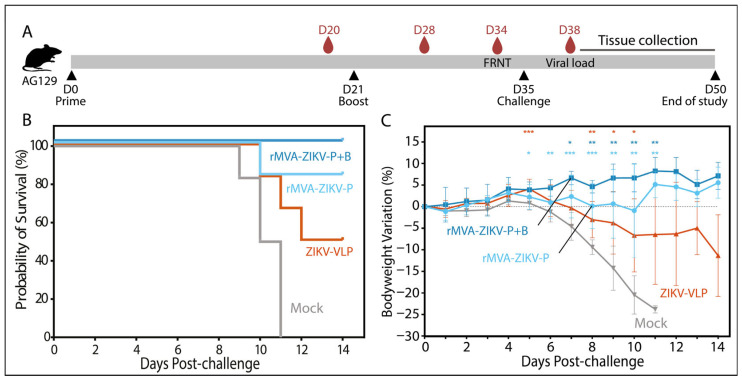
(**A**) General design of the study, (**B**) Kaplan–Meier survival curve and (**C**) percentage of bodyweight change (mean ± SD) over time from AG129 mice (*n* = 6) vaccinated with one or two doses of rMVA-ZIKV (prime only or prime & boost), ZIKV VLPs as positive control or PBS as negative control, that were then infected with 1 × 10^2^ PFU of H/PF/2013 ZIKV strain via IM route. Statistical significance was determined by a log-rank test (Mantel–Cox) for survival analysis and using a two-way ANOVA with an uncorrected Fisher post hoc analysis to compare body weight change in vaccinated animals with the negative control group at each timepoint; * *p* < 0.05, ** *p* < 0.01, *** *p* < 0.001.

To assess the immunogenicity and protective efficacy of rMVA-ZIKV, AG129 mice were immunized with either a single dose (prime) or a two-dose regimen (prime–boost) of the vaccine candidate and subsequently challenged with a lethal dose of the ZIKV H/PF/2013 strain (Figure 2A). Mice vaccinated with a virus-like particle (VLP)-based vaccine and animals receiving vaccine diluent alone (mock) were included as control groups. Immunization with rMVA-ZIKV conferred robust protection against ZIKV infection and effectively prevented infection-associated weight loss. This protective effect was superior to that observed in mice immunized with the adjuvanted VLP vaccine, which has previously been reported to confer protection in this model [14]. Although rMVA-ZIKV-vaccinated mice exhibited no significant weight loss overall, animals receiving the prime–boost regimen displayed a more stable bodyweight profile than those vaccinated with a single dose. Consistent with this observation, a survival rate of 80% was achieved following single-dose vaccination, whereas prime–boost immunization resulted in 100% survival (Figure 2B). Compared with mock-treated animals, mice receiving two doses of rMVA-ZIKV showed a significant divergence in bodyweight trajectories from day 5 post-infection onwards (Figure 2C), with vaccinated animals regaining weight as mock-treated mice developed progressive clinical disease, culminating in 0% survival by day 11 of the observation period.

### 3.3. Prime–Boost Viral Vector Vaccination Induces Higher Neutralizing Antibody Titers

**Figure 3:**
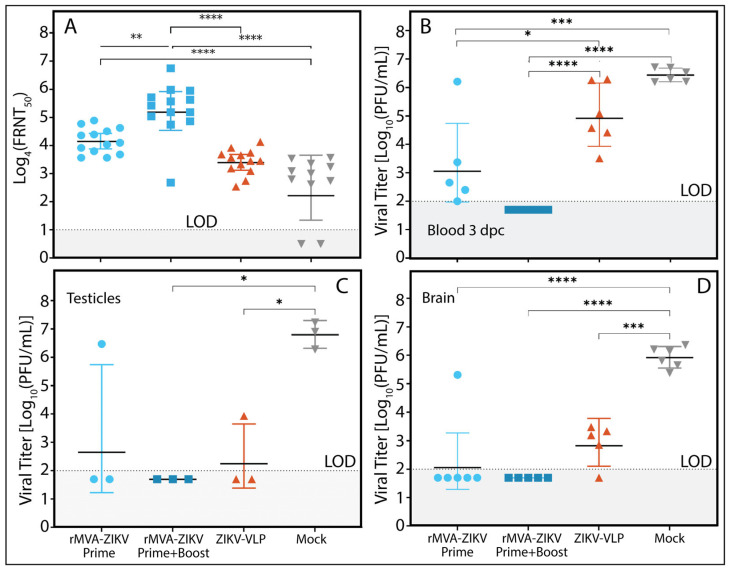
(**A**) Evaluation of the neutralizing antibody response against ZIKV strain PRVABC-59 in AG129 mice vaccinated with a single (Prime; light-blue circles) or two doses (Prime + Boost; dark-blue squares) of rMVA-ZIKV vaccine, ZIKV-VLPs (dark-orange triangles), and the negative control group (Mock; gray inverted triangles). Serum samples were collected on day 34 post-vaccination, prior to viral challenge. Results are expressed as Log-transformed FRNT_50_ titers (GM ± GSD). (**B**) ZIKV Infectious titers (GM ± GSD) detected in the blood of AG129 mice (*n* = 6) at 3 dpc. Viral titers were measured in (**C**) testicular and (**D**) brain tissues collected at the time of sacrifice, which was determined individually based on clinical signs and body weight loss. Viral titration was performed by standard plaque assay on Vero cells. Each panel includes a dashed line marking the assay’s limit of detection (LOD), and a gray-shaded region below the LOD indicating values considered negative. Statistical significance was tested using a one-way ANOVA followed by a Tukey post hoc analysis comparing means between experimental groups; * *p* < 0.05, ** *p* < 0.01, *** *p* < 0.001, **** *p* < 0.0001.

To evaluate the humoral immune response elicited by rMVA-ZIKV, neutralization assays were performed using sera collected from AG129 mice immunized with either a single dose (prime) or a two-dose regimen (prime–boost) of the vaccine. Sera were obtained on day 34 post-immunization and assessed for their ability to neutralize the ZIKV PRVABC-59 strain. As shown in Figure 3A, mice receiving the prime–boost regimen developed significantly higher neutralizing antibody titers than those vaccinated with a single dose, underscoring the critical role of booster immunization in enhancing the magnitude and quality of the humoral response. In contrast, animals vaccinated with the VLP-based control vaccine or receiving mock treatment exhibited low and comparable neutralizing activity, indicating limited humoral immunogenicity under the conditions tested.

### 3.4. rMVA-ZIKV Vaccination Reduces Viremia and Restricts Viral Dissemination to the Brain and Testes

Assessment of viremia at 3 days post-challenge (dpc) demonstrated that prime–boost vaccination with rMVA-ZIKV effectively prevented ZIKV replication during the early phase of infection. In comparison, a single vaccine dose significantly reduced viremia relative to control groups (VLP- and mock-vaccinated mice) but did not achieve complete viral suppression (Figure 3B). By the end of the observation period (2 weeks post-challenge), ZIKV was undetectable in the blood of all vaccinated animals. In contrast, mice in the mock group exhibited detectable viremia, with a geometric mean titer of 1.947 log_10_ PFU/mL (geometric standard deviation [GSD] 1.207). Moreover, rMVA-ZIKV vaccination effectively prevented viral replication in the brain and testes, two organs closely associated with severe ZIKV-associated pathology (Figure 3C,D).

### 3.5. Cell-Mediated Immunity Induced by rMVA-ZIKV Vaccination Contributes to Protection Against a French Polynesian ZIKV Strain

**Figure 4:**
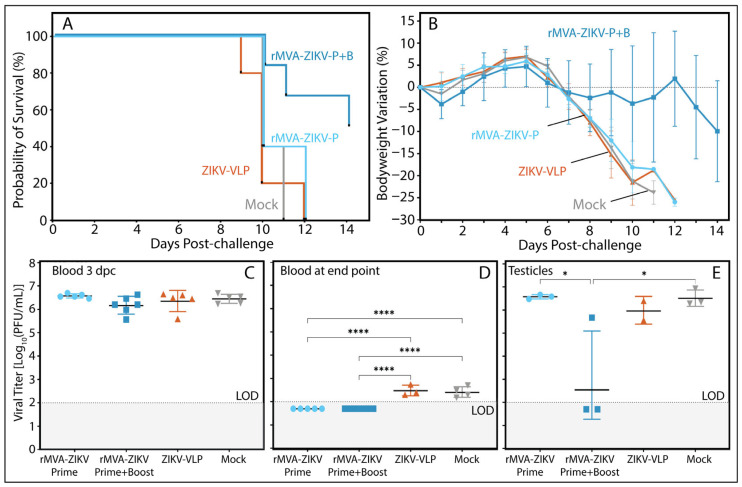
(**A**) Kaplan–Meier survival curve and (**B**) percentage of bodyweight change (mean ± SD) over time from AG129 naïve mice (*n* = 5) inoculated with splenocytes isolates from mice vaccinated. ZIKV Infectious titers (mean ± SD) were detected in the blood (**C**) 3 dpc and (**D**) during the endpoint of the study for this assessment. Viral titers were also tested in (**E**) testicular tissue collected at the time of sacrifice, which was determined individually based on clinical signs and body weight loss. The four experimental groups are shown as colored lines (panels (**A**,**B**)) and symbols (panels (**C**–**E**)), representing naïve animals receiving splenocytes from: single-dose rMVA-ZIKV (Prime; light blue), two-dose rMVA-ZIKV (Prime + Boost; dark blue), ZIKV-VLP (dark orange) and Mock (gray) vaccinated groups. Panels (**C**–**E**), include a dashed line indicating the assay’s limit of detection (LOD), with a gray-shaded region below the LOD marking values considered negative. Statistical significance was tested using a log-rank test (Mantel–Cox) for survival analysis and a two-way ANOVA with an uncorrected Fisher post hoc analysis to compare body weight change in vaccinated animals with the negative control group at each timepoint. For ZIKV infectious titers, statistical significance was tested using a one-way ANOVA followed by a Tukey post hoc analysis comparing means between experimental groups; * *p* < 0.05, **** *p* < 0.0001.

To delineate the contribution of cellular immunity to the protective efficacy observed following rMVA-ZIKV vaccination, an adoptive transfer experiment was performed in which naïve AG129 mice received splenocytes from vaccinated donors and were subsequently challenged with ZIKV. Transfer of splenocytes from mice immunized with the prime–boost rMVA-ZIKV regimen conferred significant protection against infection-associated weight loss and markedly reduced mortality compared with control groups and with recipients of splenocytes from single-dose–vaccinated animals (Figure 4). Although splenocyte transfer did not prevent viral replication during the early viremic phase at 3 days post-challenge (dpc) (Figure 4C), recipients of splenocytes from the prime–boost group exhibited enhanced control of infection at later stages. This was evidenced by reduced viral loads in blood and testicular tissue at the study endpoint (Figure 4D,E).

These findings indicate that cellular immune responses elicited by booster vaccination with rMVA-ZIKV contribute to limiting disease severity and facilitating viral clearance during late stages of ZIKV infection, although not as a fully sufficient protective mechanism in isolation.

### 3.6. Humoral Immunity Elicited by rMVA-ZIKV Vaccination Confers Protection Against a Puerto Rican ZIKV Strain

Although the protective efficacy of rMVA-ZIKV vaccination against the H/PF/2013 strain was demonstrated, most ZIKV strains currently circulating are genetically closer to viruses associated with the 2015–2016 outbreak in the Americas. We therefore investigated whether the humoral immune response induced by rMVA-ZIKV vaccination alone was sufficient to confer protection against a more recent and epidemiologically relevant strain, PRVABC-59.

**Figure 5:**
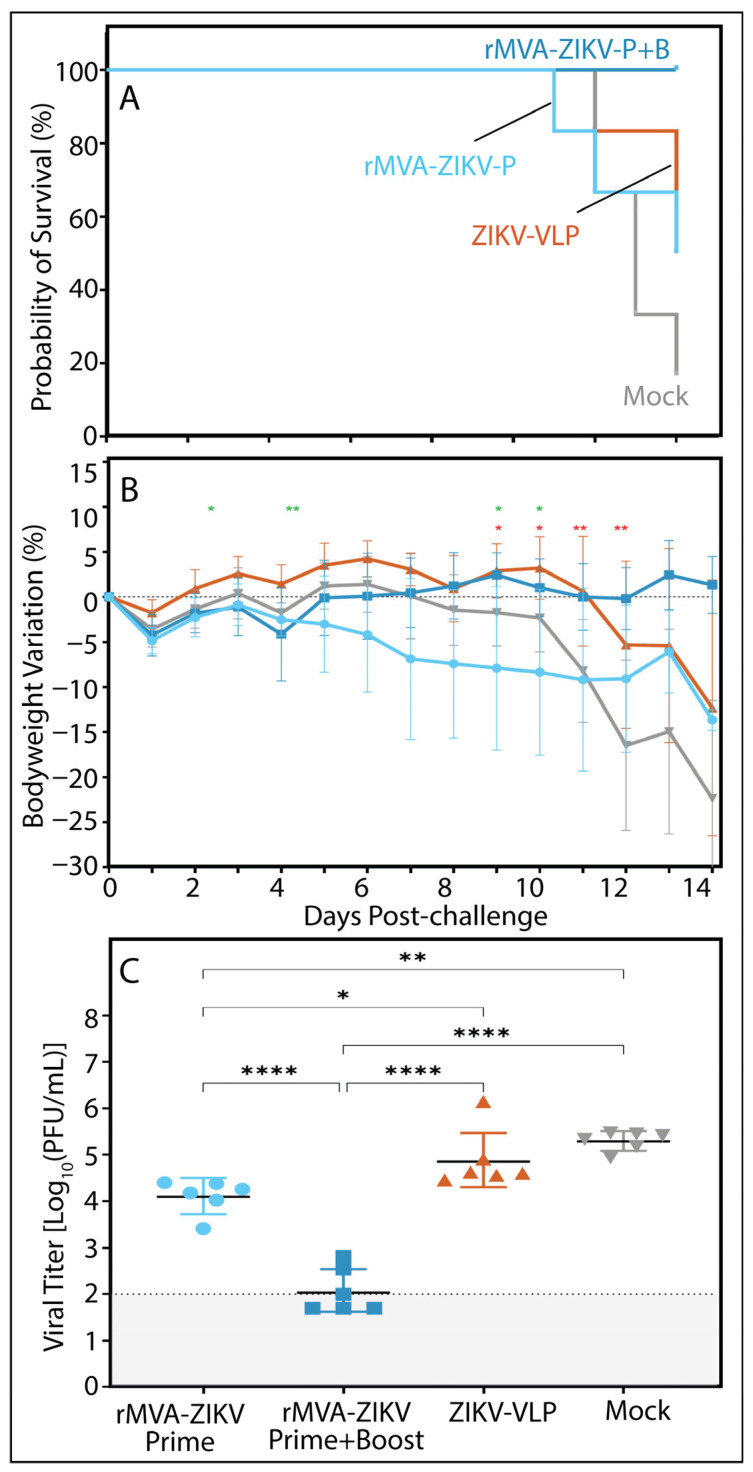
(**A**) Kaplan–Meier survival curve and (**B**) percentage of bodyweight change (mean ± SD) over time from AG129 naïve mice (*n* = 6) inoculated with blood sera collected from mice vaccinated with one or two doses of rMVA-ZIKV (prime only or prime & boost), ZIKV VLPs as positive control and PBS as negative control (mock). Sixteen hours later, animals were infected with 1 × 10^4^ PFU of PRVABC59 ZIKV strain via the IP route. (**C**) ZIKV infectious titers (mean ± SD) were evaluated in the blood collected 3 days post-challenge. The four experimental groups are shown as colored lines (panels A and B) and symbols (panel C), representing naïve animals receiving blood sera from: single-dose rMVA-ZIKV (Prime; light blue), two-dose rMVA-ZIKV (Prime + Boost; dark blue), ZIKV-VLP (dark orange) and Mock (gray) vaccinated groups. Panel C, includes a dashed line indicating the assay’s limit of detection (LOD), with a gray-shaded region below the LOD marking values considered negative. Statistical significance was tested using a log-rank test (Mantel–Cox) for survival analysis, a two-way ANOVA with an uncorrected Fisher post hoc analysis to compare body weight change in vaccinated animals with negative control group on each timepoint and an one-way ANOVA followed by a Tukey post hoc analysis comparing means between experimental groups for viremia determination; * *p* < 0.05, ** *p* < 0.01, **** *p* < 0.0001.

Passive transfer of sera from mice immunized with the prime–boost rMVA-ZIKV regimen protected naïve recipients from infection-associated weight loss and lethality following challenge with PRVABC-59 (Figure 5A,B). In addition, serum transfer significantly reduced viremia during the early phase of infection at 3 days post-challenge (dpc), indicating effective control of viral replication during the acute viremic stage (Figure 5C). Histopathological examination of the brain, testes, liver, kidney, and spleen revealed no major differences across experimental groups; however, the most pronounced lesions were observed in the brain and testes. Notably, animals receiving the prime–boost rMVA-ZIKV sera exhibited reduced severity of brain tissue damage compared with other groups. Detailed histopathological findings are provided in the Supplementary Information (Figure S2).

These results demonstrate that rMVA-ZIKV vaccination induces a potent humoral immune response capable of mediating protection against heterologous ZIKV strains. This antibody-mediated protection is likely driven by neutralizing antibodies targeting key epitopes within ZIKV structural proteins involved in host cell attachment and entry.

## 4. Discussion

Following the Zika virus outbreak in 2015–2016, a spectrum of severe adverse outcomes was identified, most notably congenital Zika syndrome (CZS), characterized by microcephaly and other profound central nervous system abnormalities in newborns, and Guillain–Barré syndrome in adults [17,18]. These complications underscored the substantial public health impact of ZIKV infection and established the virus as a re-emerging threat with significant epidemic potential. Although large outbreaks have not recurred at the same scale, the continued circulation of ZIKV and the presence of competent vectors sustain the risk of future epidemics, reinforcing the need for effective and safe vaccines as a core component of preparedness strategies.

In this study, we evaluated a viral-vectored vaccine based on Modified Vaccinia Ankara (MVA) expressing highly conserved ZIKV pre-membrane (prM) and envelope (E) antigens derived from a consensus sequence representative of the Asian lineage responsible for recent epidemics [19,20]. Following successful production and molecular characterization of rMVA-ZIKV, we assessed the protective efficacy of single-dose and prime–boost regimens in AG129 mice, a well-established model for ZIKV pathogenesis and vaccine evaluation [10].

In preclinical challenge studies, rMVA-ZIKV vaccination conferred 80% protection against lethal challenge with the H/PF/2013 strain following a single dose and 100% protection following a prime–boost regimen, accompanied by favorable clinical outcomes, including prevention of infection-associated weight loss. These findings are operationally relevant for outbreak scenarios, in which rapid induction of protective immunity is critical and adherence to multi-dose vaccination schedules may be logistically challenging [21,22]. The observed efficacy aligns with previous reports showing that MVA-based ZIKV vaccines expressing prM/E antigens elicit neutralizing antibody responses and reduce viremia in susceptible, interferon-deficient mouse models. These findings also build on earlier studies in which limited efficacy required multiple dosing or the use of adjuvantation strategies [23,24]. Moreover, the robust neutralizing antibody titers elicited by our vaccine against a heterologous ZIKV strain (PRVABC-59) provide preliminary evidence supporting its potential to induce broad antigenic coverage. These findings underscore the effectiveness of the antigen design strategy in eliciting immune responses with the potential to target diverse ZIKV antigenic variants. Future studies should expand upon these results by systematically evaluating neutralization breadth across multiple genetically distinct ZIKV strains to more comprehensively define the scope of vaccine-induced protective immunity.

Analysis of serum viral loads at three days post-challenge revealed no detectable systemic ZIKV replication in animals receiving two doses of rMVA-ZIKV. Although mice vaccinated with a single dose also exhibited a marked reduction in viremia compared with VLP- and mock-vaccinated controls, low-level viral replication remained detectable, indicating that boosting substantially enhances early containment of infection. By the study endpoint, infectious virus was undetectable in all vaccinated groups, whereas mock-treated animals remained viremic, highlighting the capacity of MVA-based vaccination to accelerate and deepen protective immune responses.

A similar pattern was observed in key target organs. In the prime–boost rMVA-ZIKV group, viral RNA was undetectable in both the brain and testes of all evaluated animals. In contrast, among single-dose recipients, at least one animal exhibited high viral loads in both tissues, while others remained virus-negative. These findings suggest that booster vaccination prevents the establishment of viremia and consequently blocks tissue-specific viral replication, promoting complete viral clearance. In contrast, single-dose vaccination, while insufficient to fully prevent late-stage tissue infection, nonetheless induces immune responses capable of substantially limiting viral replication. The VLP-vaccinated group displayed partial protection, with reduced tissue viral loads compared with mock-treated animals, which exhibited consistently high titers. Preventing viral dissemination to critical organs is particularly important in the context of ZIKV infection, as neurological involvement is strongly associated with disease severity [25]. Clinical manifestations, including encephalopathy, encephalitis, meningitis, myelitis, and seizures, have been reported, supporting a direct neuropathogenic role of the virus [26]. Moreover, ZIKV infection of the testes may result in testicular atrophy and prolonged viral persistence in reproductive tissues and semen, contributing to sexual transmission [27]. Together, these observations suggest that a two-dose rMVA-ZIKV regimen may not only prevent mortality but also reduce secondary complications and limit sexual transmission by blocking viral persistence in reproductive organs.

Mechanistic studies further elucidated potential immune correlates of protection. Neutralizing antibody titers measured before challenge were significantly higher in the prime–boost group than in single-dose or control groups, consistent with rapid control of viremia at three days post-challenge and with previous studies linking neutralizing antibody levels to protection in AG129 mice and non-human primates [9]. Passive transfer experiments reinforced the central role of humoral immunity: pooled sera from prime–boost–vaccinated mice resulted in 100% survival following PRVABC59 challenge, with full recovery of bodyweight, whereas sera from single-dose rMVA-ZIKV and VLP-vaccinated mice conferred partial protection (>60% and 50% survival, respectively) and were associated with transient weight loss. These findings indicate that optimal antibody-mediated protection against ZIKV requires a prime–boost vaccination strategy.

The PRVABC59 strain was selected as a contemporary challenge virus due to its circulation during the American outbreak and its association with adverse pregnancy outcomes in epidemiological and experimental studies [28]. This allowed assessment of vaccine-induced cross-protection against strains with ongoing epidemic relevance. The prM and E antigens included in the vaccine are well-established targets of neutralizing antibodies that cross-react across ZIKV strains, supporting the potential for heterologous protection [8,29]. Nevertheless, given the dynamic evolution of viral epitopes, continued evaluation of cross-neutralization breadth remains essential.

The contribution of cellular immunity was explored through adoptive transfer of splenocytes. Transfer from prime–boost rMVA-ZIKV donors conferred 50% survival following H/PF/2013 challenge, whereas all control animals succumbed to infection within 12 days. Notably, adoptively transferred cellular immunity did not prevent early viremia entirely, suggesting that cellular mechanisms function optimally when integrated with humoral immunity rather than as standalone protective factors. These results align with previous MVA-based ZIKV studies demonstrating robust CD8^+^ T cell responses and suggest that both humoral and cellular immunity contribute to protection, with antibodies mediating early viral control and T cells limiting viral dissemination and promoting clearance [23,30]. In immunocompetent models, ZIKV-specific CD8^+^ T cells have been shown to traffic to infected brain tissue, supporting a biological role for cellular immunity even when neutralizing antibodies dominate protective correlates [31]. Furthermore, type I interferon signaling has been shown to support B cell, plasma cell, and T follicular helper responses following MVA vaccination, providing a mechanistic basis for the enhanced humoral immunity observed with prime–boost regimens [32].

The robust protection observed with passive serum transfer and the more limited protection from adoptive cellular transfer provide potential mechanistic insights into the temporal requirements of protective immunity induced by our vaccine candidate. Passively transferred neutralizing antibodies can establish near-immediate immune protection during the critical early phase of infection, preventing or significantly attenuating initial viral dissemination [33]. In contrast, adoptively transferred cellular immunity requires time for T cell activation, proliferation, and differentiation into effector populations, a process that occurs during active viral replication rather than before pathogen exposure. Collectively, these findings suggest that rMVA-ZIKV-mediated protection is not driven by cellular immunity as an independent mechanism of protection. Instead, the protective efficacy of our vaccine requires a coordinated interplay between humoral and cellular immune responses that restrict early viral replication and facilitate efficient viral clearance, with neutralizing antibodies serving as the dominant protective mechanism. These mechanistic insights converge with emerging reports on ZIKV vaccine development using the MVA platform, which similarly identify antibody-mediated protection as primary, although detailed kinetic analysis dissecting humoral versus cellular contributions remains incomplete [23,34].

The AG129 mouse model has played a critical role in the study of ZIKV pathogenesis and assessment of vaccine-induced protective immunity, as its dual deficiency in type I and type II interferon signaling permits robust viral replication and lethal disease that recapitulates key features of severe human infection [10]. Multiple vaccine studies have successfully utilized this model to demonstrate protective efficacy against mortality and disease following homotypic and heterotypic viral challenge [14,35]. Importantly, the heightened susceptibility of AG129 mice allows for the differentiation of vaccine candidates that would be difficult to assess in immunocompetent models, where ZIKV infection typically results in mild or subclinical outcomes [36]. Comparative evaluations across mouse models using the same vaccine platforms indicate that immunogenicity patterns and protective mechanisms identified in AG129 mice often correlate with findings subsequently observed in partially or fully immunocompetent systems, supporting the predictive value of this model [37,38]. Nevertheless, the absence of functional interferon pathways introduces inherent limitations. Disruption of these immune signaling pathways may alter antigen presentation dynamics, T-cell priming kinetics, or the antibody maturation process, potentially limiting translational interpretation of certain immunological features to human hosts with intact interferon responses. Accordingly, our findings presented here should be interpreted as evidence of vaccine efficacy in a highly permissive and stringent infection context, rather than as a complete characterization of the full spectrum of immune responses elicited by vaccination. Future studies employing immunocompetent mouse models with temporally controlled interferon blockage or complementary non-human primate studies would provide essential validation of cellular immunity mechanisms and antibody quality observed in more physiologically relevant settings.

Our vaccine candidate, based on the MVA vector platform, offers distinct advantages for rapid vaccine development and deployment during ZIKV outbreaks: The platform functions as a modular system capable of encoding diverse pathogen-specific antigens while maintaining strong immunogenicity. The inherent adaptability of MVA, enabling antigen redesign through straightforward molecular cloning and production in avian cell lines within weeks, positions it as an attractive approach for accelerated responses to emerging viral threats [39]. Recent multi-pathogen applications exemplify this modularity: trivalent MVA vaccines encoding antigens from three district encephalitis viruses have demonstrated protective efficacy against all targets simultaneously [40], and integrated MVA platforms targeting filoviruses and arenaviruses have successfully advanced through clinical development [41]. Together, these precedents show that MVA can accommodate multiple antigenic targets without compromising immunostimulatory capacity and highlight the feasibility of rapidly redesigning our ZIKV vaccine candidate if evolving ZIKV circulation dynamics could necessitate updated or expanded antigenic coverage.

Finally, from an operational perspective, the MVA platform combines a well-established safety profile with an existing global manufacturing infrastructure (shared with adenoviral vector manufacturing development principles) that supports accelerated clinical translation [42]. The vector’s inability to replicate in mammalian cells, together with decades of clinical precedent, substantially reduces regulatory barriers compared to novel platforms, while thermal stability and simplified cold-chain requirements enhance accessibility in resource-limited settings where ZIKV transmission remains endemic. While our current rMVA-ZIKV vaccine candidate requires a two-dose prime–boost regime, emerging evidence indicates that optimized MVA formulations and alternative delivery strategies can support single-dose protection in emergency scenarios [43]. However, the value of the MVA platform for outbreak preparedness does not lie in providing a standalone universal solution, but rather in serving as a key component within diversified vaccine portfolios. In such integrated strategies, MVA-based vaccines provide critical redundancy and manufacturing flexibility. Indeed, pandemics preparedness frameworks increasingly recognize that platform diversification, rather than single-technology reliance, enables rapid, equitable, and resilient outbreak responses [44,45]. In this integrated context, the rMVA-ZIKV candidate could represent a readily available, technologically proven modular vaccine component capable of supporting rapid deployment alongside mRNA-based and subunit vaccine approaches to address ZIKV reemergence.

## 5. Conclusions

Our findings support the continued development of MVA-based ZIKV vaccines as a promising preventive strategy for outbreak preparedness and disease prevention. The rMVA-ZIKV candidate exhibited a favorable safety profile and elicited strong protective efficacy in a stringent challenge preclinical model using a two-dose prime–boost regimen. Importantly, vaccination elicited strong neutralizing antibody responses capable of targeting a heterologous ZIKV strain, highlighting the breadth of the induced humoral immunity. Passive transfer studies underscored the central role of vaccine-induced antibodies in mediating protection, providing direct functional evidence that the humoral response is a key correlate of immunity in this model. In addition, although to a lesser extent, adoptive transfer experiments revealed contributions of the cellular immune response, which likely acts synergistically with the more dominant antibody-mediated mechanisms to achieve the observed protective profile. Together, these results underscore the immunological robustness and versatility of the MVA platform and provide compelling justification to support rMVA-ZIKV advancement toward translational development and subsequent clinical evaluations as a countermeasure against future ZIKV outbreaks.

## Data Availability

The authors declare that the data supporting the findings of this study are available within the paper and Supplementary Materials. Further information is available from the corresponding authors upon reasonable request.

## Supplementary Materials

The following supporting information can be downloaded at https://www.mdpi.com/article/10.3390/vaccines14030252/s1, Figure S1. Generation of the rMVA-ZIKV vaccine candidate; Figure S2. Multi-tissue histopathological findings in serum-transferred AG129 mice challenged with ZIKV.
