# Supplementary material for: Protective Efficacy of a Modified Vaccinia Ankara-Based Vaccine Against Zika
Source: Vaccines (Basel). 2026 Mar 10;14(3):252. doi: 10.3390/vaccines14030252 (PMC13030437; doi:10.3390/vaccines14030252)
Supplement: Supplementary file 1 [file vaccines-14-00252-s001.zip › Supplementary information.pdf]

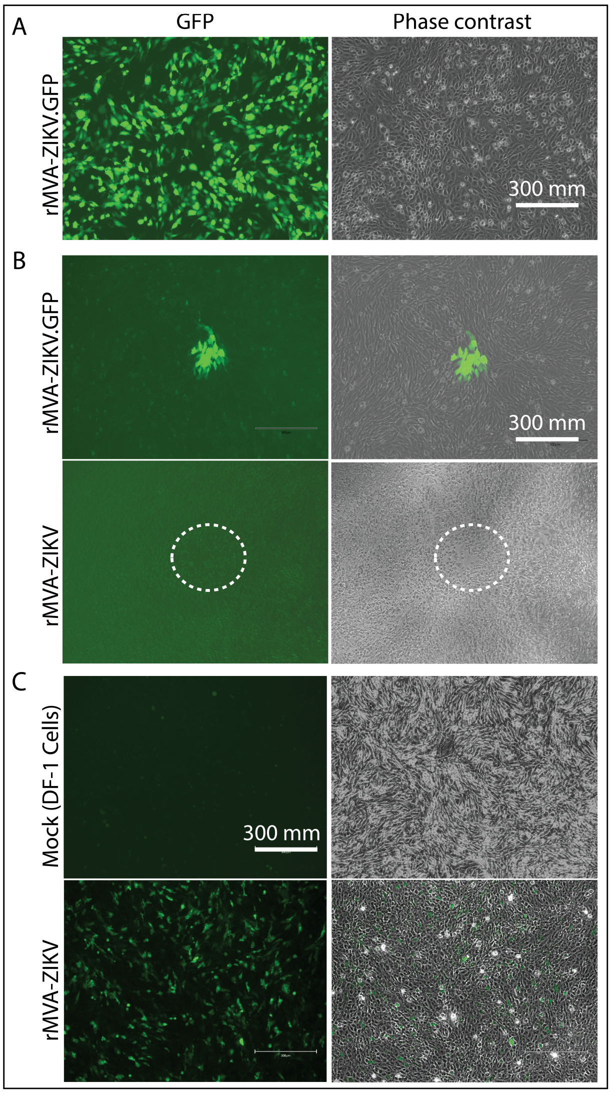

Figure S1. Generation of the rMVA-ZIKV vaccine candidate. **A.** GFP fluorescence observed in DF-1 cells 24 hours after transfection with the plasmid containing the target sequence for rMVA-ZIKV.GFP. **B** Confirmation of the rMVA-ZIKV clone lacking fluorescence in DF-1 cells; cytopathic effect is evident, but no fluorescence is detected under the microscope. **C**. Verification of envelope protein expression in DF-1 cells from the rMVA-ZIKV stock. Fluorescent cells indicate E protein expression, alongside a control of non-infected cells.

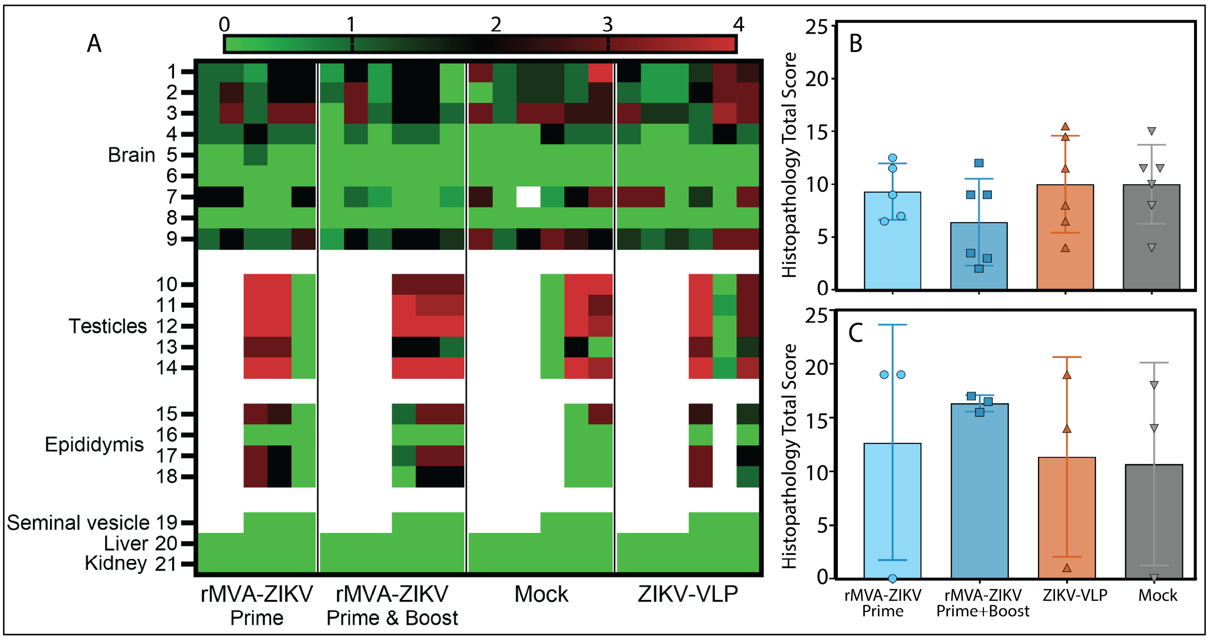

Figure S2. (**A**) Heatmap illustrating individual histopathology scores (ranging from 0: no observable lesions to 4: severe histopathological findings) for each animal across evaluated tissue-specific parameters. **Brain:** (1) neuronal degeneration, necrosis or apoptosis; (2) inflammation in areas of necrosis (neutrophilic and lymphoplasmacytic); (3) perivascular inflammation; (4) vascular fibrinoid necrosis; (5) calcification; (6) ventriculomegaly; (7) hippocampal degeneration/apoptosis; (8) cerebellar degeneration/apoptosis; and (9) meningeal inflammation. **Testicles:** (10) necrosis; (11) inflammation; (12) degeneration of seminiferous tubules; (13) fibrosis; (14) lymphocytic, histiocytic, or neutrophilic orchitis. **Epididymis:** (15) inflammatory infiltration; (16) lymphoid follicle formation; (17) epithelial cell regeneration, and (18) peritubular fibrosis. **Seminal vesicle:** (19) coagulating gland lesion. **Liver:** (20) pathological finding/lesion. **Kidney:** (21) pathological finding/lesion. Data represents AG129 naïve mice (n = 5) inoculated with blood sera collected from the immunogenicity and efficacy study, followed by infection with 1×10^4 PFU of the PRVABC59 ZIKV strain via intraperitoneal (IP) route, as previously described. Panels (B) and (C) show total histopathology scores (mean ± SD) for brain and testicular tissues, respectively. A non-parametric Kruskal–Wallis test was used to compare total score distributions between experimental groups.
