# Supplementary figures and images for: Protective Efficacy of a Modified Vaccinia Ankara-Based Vaccine Against Zika
Source: Vaccines (Basel). 2026 Mar 10;14(3):252. doi: 10.3390/vaccines14030252 (PMC13030437; doi:10.3390/vaccines14030252)

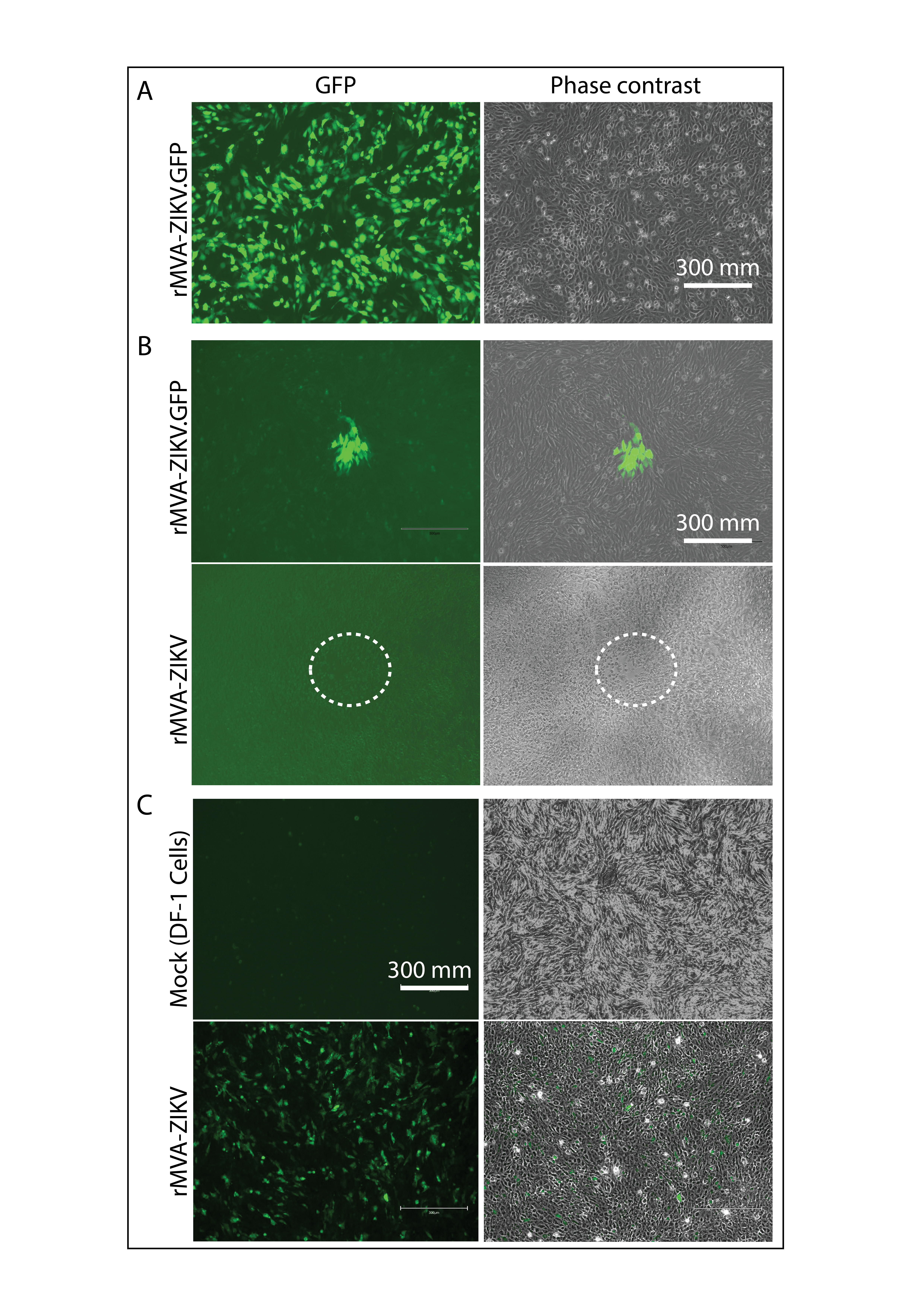

Supplement: Supplementary file 1 [file vaccines-14-00252-s001.zip › FigureS1.png]

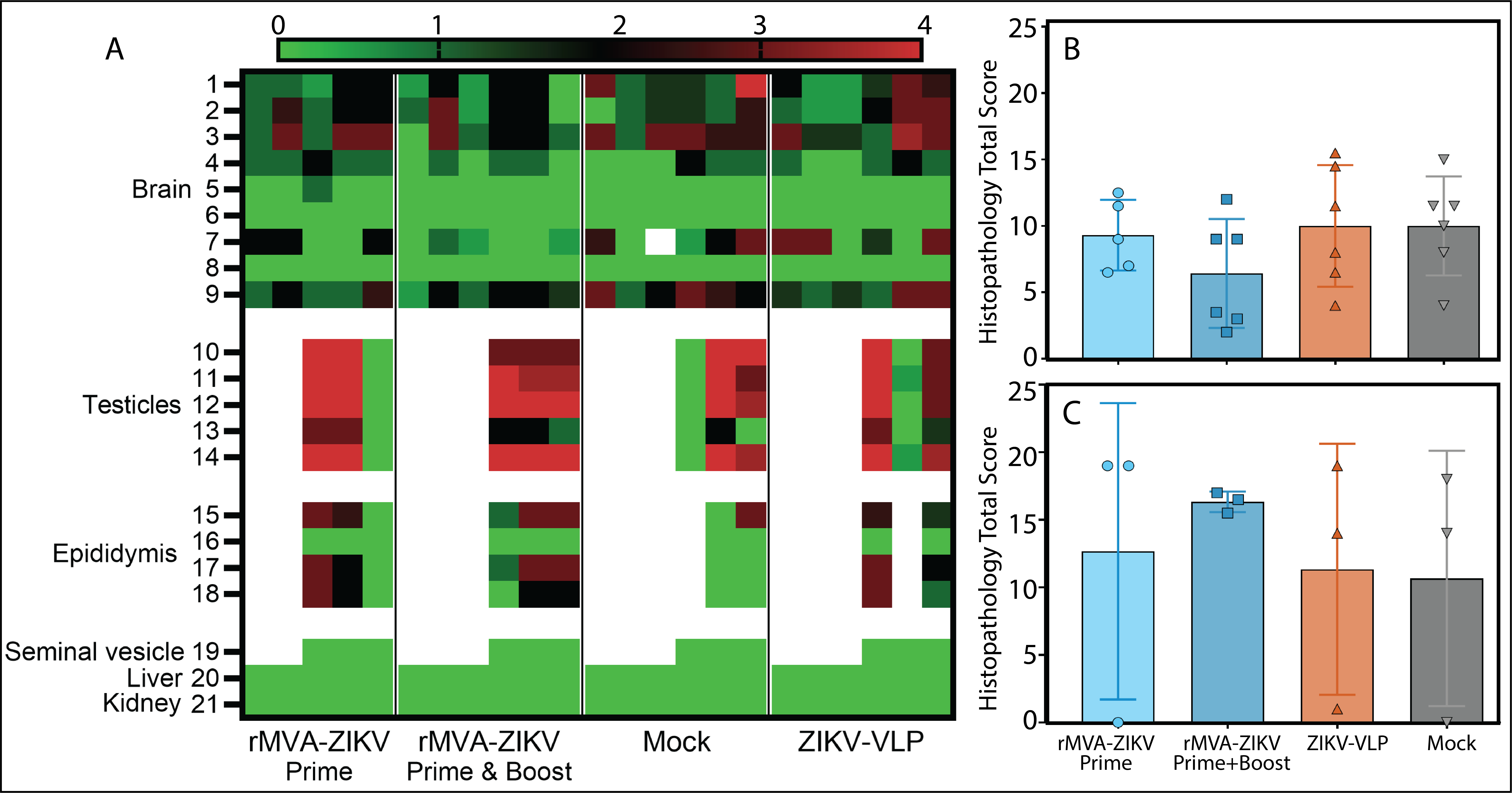

Supplement: Supplementary file 1 [file vaccines-14-00252-s001.zip › FigureS2.png]
